# Endovascular thrombectomy is cost-saving in patients with acute ischemic stroke with large infarct

**DOI:** 10.3389/fneur.2024.1324074

**Published:** 2024-04-17

**Authors:** Julian Schwarting, Matthias F. Froelich, Jan S. Kirschke, Dirk Mehrens, Jannis Bodden, Dominik Sepp, Jonas Reis, Konstantinos Dimitriadis, Jens Ricke, Claus Zimmer, Tobias Boeckh-Behrens, Wolfgang G. Kunz

**Affiliations:** ^1^Department of Diagnostic and Interventional Neuroradiology, TUM School of Medicine, Technical University Munich, Munich, Germany; ^2^Department of Radiology/Neuroradiology, Berufsgenossenschaftliche Unfallklinik, Murnau Am Staffelsee, Germany; ^3^Institute for Stroke and Dementia Research (ISD), LMU Munich University Hospital, Munich, Germany; ^4^Department of Radiology and Nuclear Medicine, University Medical Centre Mannheim, University of Heidelberg, Mannheim, Germany; ^5^Department of Radiology, LMU University Hospital, Munich, Germany; ^6^Institute of Neuroradiology, LMU University Hospital, LMU Munich, Munich, Germany

**Keywords:** stroke, cost-effectiveness, thrombectomy, endovascular treatment, ASPECT score

## Abstract

**Objective:**

Endovascular thrombectomy (EVT) is the standard of care for acute large vessel occlusion stroke. Recently, the ANGEL-ASPECT and SELECT 2 trials showed improved outcomes in patients with acute ischemic Stroke presenting with large infarcts. The cost-effectiveness of EVT for this subpopulation of stroke patients has only been calculated using data from the previously published RESCUE-Japan LIMIT trial. It is, therefore, limited in its generalizability to an international population. With this study we primarily simulated patient-level costs to analyze the economic potential of EVT for patients with large ischemic stroke from a public health payer perspective based on the recently published data and secondarily identified determinants of cost-effectiveness.

**Methods:**

Costs and outcome of patients treated with EVT or only with the best medical care based on the recent prospective clinical trials ANGEL-ASPECT, SELECT2 and RESCUE-Japan LIMIT. A A Markov model was developed using treamtment outcomes derived from the most recent available literature. Deterministic and probabilistic sensitivity analyses addressed uncertainty.

**Results:**

Endovascular treatment resulted in an incremental gain of 1.32 QALYs per procedure with cost savings of $17,318 per patient. Lifetime costs resulted to be most sensitive to the costs of the endovascular procedure.

**Conclusion:**

EVT is a cost-saving (i.e., dominant) strategy for patients presenting with large ischemic cores defined by inclusion criteria of the recently published ANGEL-ASPECT, SELECT2, and RESCUE-Japan LIMIT trials in comparison to best medical care in our simulation. Prospective data of individual patients need to be collected to validate these results.

## Introduction

Stroke remains despite significant advances in therapy the leading cause for long-term disability (1). Endovascular therapy (EVT) was established as standard therapy for large vessel occlusion stroke over the last years and has demonstrated effectiveness in comparison to best medical care (BMC). However, patients with large ischemic strokes, classified by large infarct cores, have been underrepresented in previous prospective studies because of suspected increased risk of intracerebral hemorrhage after revascularization (2).

This has recently changed with the publication of the Japanese RESCUE-Japan LIMIT trial (3) in 2022 and the ANGEL-ASPECT (4) and SELECT2 (5) trials in 2023, which demonstrated the superiority of endovascular treatment of stroke patients with large infarcts.

Endovascular recanalization of large-vessel occlusions in the anterior circulation has been shown to lead to extensive cost-savings for worldwide healthcare systems in the long-term (6–9). Cost-effectiveness for the investigated subgroup of patients presenting with large ischemic infarcts was shown based on data from the RESCUE-Japan trial (10).

Because the generalizability of this data to an international population is limited, this study aims to quantify and compare lifetime benefits and direct healthcare costs of patients with large ischemic stroke caused by anterior circulation large vessel occlusion based on a pooled analysis of the recently published prospective trials.

## Methods

### Study selection

The simulation of long-term cost differences and outcomes of patients was based on the recently published international multicenter, prospective, randomized controlled trials ANGEL-ASPECT (5) SELECT2 (4), and RESCUE-Japan LIMIT (3) which are summarized in Table 1.

**Table 1:** Input studies.

	ANGEL-ASPECT (5)	SELECT2 (4)	RESCUE-Japan LIMIT (3)
Inclusion time	2020–2022	2019–2022	2018–2021
Age	18 – 80y	18 – 85y	≥ 18y
Country	China	International	Japan
**Inclusion criteria**			
Affected vessel	Prox. M1 or ICA	Prox. M1 or ICA	M1 or ICA
mRS	0–1	0–1	0–1
NIHSS	6–30	≥6	≥6
Infarct demarcation	*ASPECTS* 3–5 **or** *ASPECTS* > 5 (6 h-24 h); infarct core volume 70–100 mL **or** *ASPECTS* < 3; infarct core volume 70–100 mL **and** No mass effect	*ASPECTS* 3–5 **or** > 50 mL* and No mass effect	*ASPECTS* 3–5 **or** *DWI-ASPECTS* 3–5 **and** No mass effect
Symptom onset to inclusion	< 24 h	< 24 h	0–6
**Study participants**			
Screened for participation	1,504	958	235
Total participants	456	352	203
Intervention group	231	178	100
**Intravenous thrombolysis**			
EVT	29%	21%	27%
BMC	28%	17%	28%

Model input	Base-case value	Distribution	Source
Assumed starting age	67 years	-	ANGEL-ASPECT (4), SELECT2 (4), and RESCUE-Japan LIMIT (3)
Discount rate	3.00%	–	
**Cost**			
Stroke hospitalization costs (mRS 0–2 / 3–5 / 6)	$26,705 / $106,533 / $88,159	γ	Mu (13)
Costs of first 90 days after discharge (mRS 0–2 / 3–5)	$15,213 / $26,496	γ	
Total costs day 91–365 after stroke (mRS 0–2 and 3–5)	$24,988 / $35,140	γ	
Additional cost EVT	$17,103	γ	Shireman (9)
Acute care costs recurrent stroke	$29,259	γ	Kunz (14)
Long-term annual cost after stroke for mRS 0 / 1 / 2 / 3 / 4 / 5	$14,230 / $14,653 / $16,952 / $29,107 / $58,913 / $86,612	γ	Shireman (9)
**Initial probabilities**			
Functional outcome 90d after EVT mRS 0 / 1 / 2 / 3 / 4 / 5 / 6 in %	3.9 / 8.3 / 17.8 / 17.0 / 19.6 / 11.7 / 21.7	-	ANGEL-ASPECT (5)
Functional outcome 90d after BMC mRS 0 / 1 / 2 / 3 / 4 / 5 / 6 in %	0 / 0 / 3.6 / 21.8 / 26.7 / 20.0 / 20.0	-	
Functional outcome 90d after EVT mRS 0 / 1 / 2 / 3 / 4 / 5 / 6 in %	1.1 / 5.1 / 14.0 / 17.4 / 15.2 / 8.4 / 38.2	-	SELECT2 (4)
Functional outcome 90d after BMC mRS 0 / 1 / 2 / 3 / 4 / 5 / 6 in %	0 / 1.7 / 5.2 / 11.5 / 20.7 / 18.4 / 40.8	-	
Functional outcome 90d after EVT mRS 0 / 1 / 2 / 3 / 4 / 5 / 6 in %	2.0 / 3.0 / 9.0 / 17.0 / 33.0 / 18.0 / 18.0	-	RESCUE-Japan LIMIT (3)
Functional outcome 90d after BMC mRS 0 / 1 / 2 / 3 / 4 / 5 / 6 in %	0 / 3.0 / 5.0 / 5.0 / 25.0 / 40.0 / 24.0	-	
Functional outcome 90d after EVT mRS 0 / 1 / 2 / 3 / 4 / 5 / 6 in %	2.7 / 6.9 / 16.2 / 17.2 / 17.7 / 10.3 / 29.0		Weighted means of all included studies
Functional outcome 90d after BMC mRS 0 / 1 / 2 / 3 / 4 / 5 / 6 in %	0 / 2.8 / 6.8 / 17.4 / 24.2 / 19.4 / 29.3		

### Economic model

**Figure 1:**
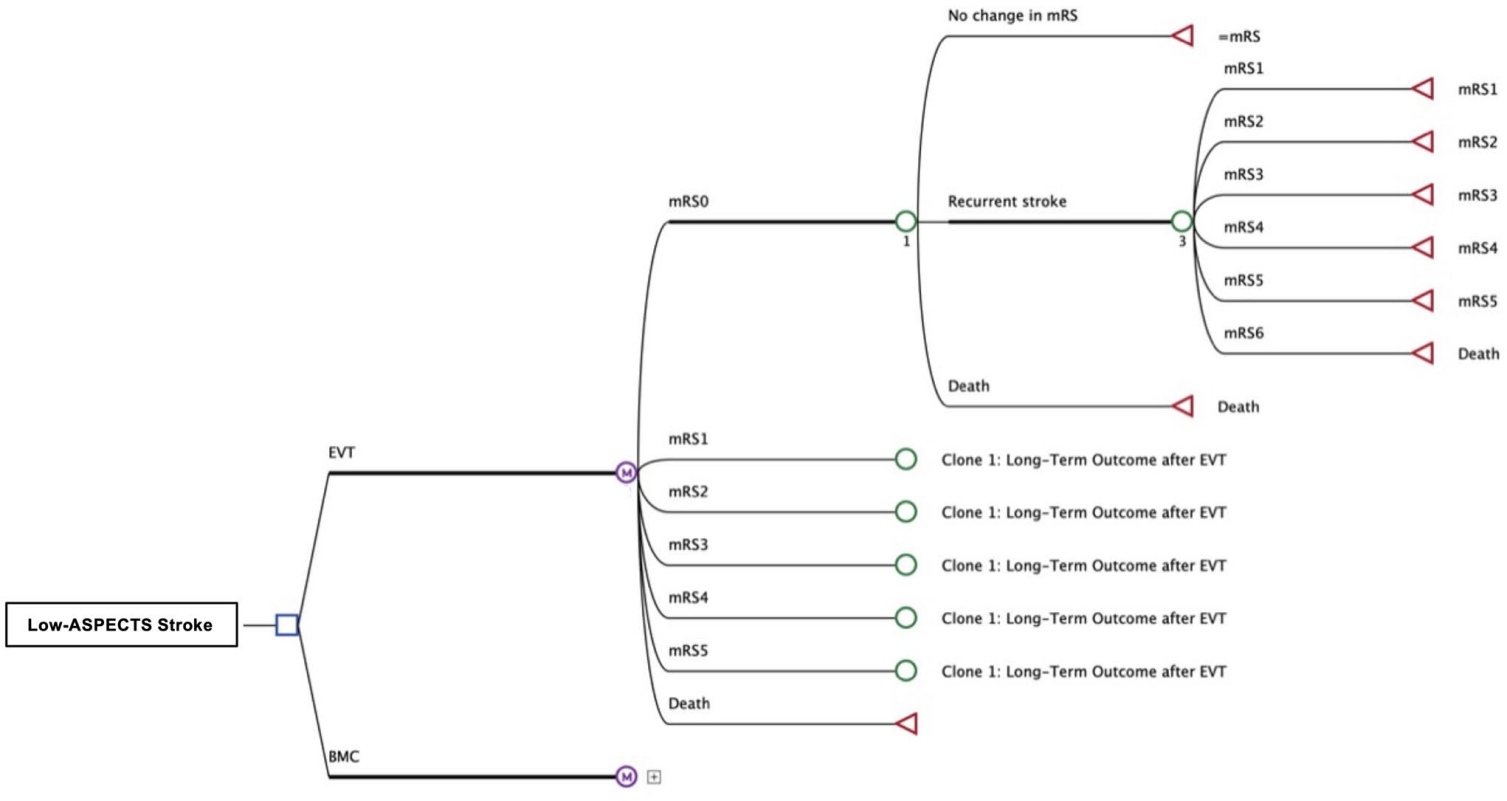
Structure of Decision Tree and Markov-Model Patients with occlusion of a large anterior circulation artery and an ASPECT score of 3–5 received either the best medical care (BMC) or endovascular thrombectomy (EVT). The adjacent Markov model simulates the lifetime pathways of patients after stroke with possibly reduced functional independence, according to outcomes reported after both treatment strategies. Costs and effectiveness (QALYs) were compared for EVT and BMC.

Using a Markov decision model with a life-time horizon and a cycle length of 1 year we examined the disparities of quality-adjusted life years (QALYs) and costs for healthcare providers in the United States in compliance with the Consolidated Health Economic Evaluation Reporting Standards (CHEERS) (Figure 1) (11). Economic analysis was carried out using the decision-analytic software TreeAge Pro 2022 (TreeAge, Williamstown, MA, United States).

Starting point of the simulation was set immediately before endovascular thrombectomy (EVT) or best medical care (BMC) in accordance with the inclusion parameters of the primary input studies ANGEL-ASPECT (5) SELECT2 (4), and RESCUE-Japan LIMIT (3) (Table 1). The inclusion age was 67, which was the average age of the input trials. In the decision model, we differentiated patients with acute-onset large vessel occlusion into 2 cohorts (EVT or BMC). Except for the initial intervention, we assumed the same treatment and care for both cohorts.

We simulated patients’ long-term outcomes that was categorized on the level of disability reported after 90 days using the modified Rankin Scale (mRS). We allowed outcome deterioration by an occurrence of further strokes, resulting in the same or a lower mRS state and stratified mortality based on age and outcome (Figure 1).

### Model input parameters

For the simulation of costs and effectiveness, we included the prospective clinical trials ANGEL-ASPECT, Select2, and RESCUE-Japan LIMIT. Key characteristics of these studies are summarized in Table 2.

**Table 2:** Input variables.

Transition probabilities			
Stroke recurrence (year 1–10)	0.059 / 0.036 / 0.025 / 0.022 / 0.022 / 0.027 / 0.027 / 0.023 / 0.028 / 0.016	–	Pennlert (15)
Annual death hazard ratios for survivors mRS 0 / 1 / 2 / 3 / 4 / 5	0.129 / 0.136 / 0.164 / 0.247 / 0.135 / 0.189	–	Hong (16)
Outcome after recurrent stroke in mRS 0 or 1 / 2 / 3 / 4 / 5 / 6	0.129 / 0.136 / 0.164 / 0.247 / 0.135 / 0.189	–	Goyal (17)
Age-adjusted mortality	U.S. life tables	–	Arias (18)
**Utilities**			
Quality of life (mRS Score 0–5)	1.00/0.91/0.76/0.65/0.33/0.00/0.00	β	Chaisinanukul (19)

Parameters utilized in this study were derived from literature according to the recommendations of cost-effectiveness analyses. All input variables are shown in Table 3 (11, 12).

**Table 3:** Cost-effectiveness analysis.

	**Cohort**	**Costs**	**Incr. Costs**	**Effectiveness (QALY)**	**Incr. Effectiveness** **(QALY)**	**ICER** **($/QALY)**
**Best Medical Care**	*Total*	$403,044	–	2.44	–	–
	*ANGEL-ASPECT*	$420,443	–	3.25	–	–
	*SELECT2*	$336,473	–	2.06	–	–
	*RESCUE-Japan LIMIT*	$444,711		1.98		
**Endovascular thrombectomy**	*Total*	$385,726	- $17,318	3.76	1.32	Cost saving
	*ANGEL-ASPECT*	$382,679	- $37,763	4.38	1.12	Cost saving
	*SELECT2*	$325,753	- $8,610	3.31	1.25	Cost saving
	*RESCUE-Japan LIMIT*	$446,856	$2,145	3.58	1.61	$1,336/QALY

### Initial and transition probabilities

Initial functional outcome data after 90d was directly taken from the primary data published in ANGEL-ASPECT, SELECT2 and RESCUE-Japan LIMIT. We performed a separate cost-effectiveness-analysis for each of the input studies. For the combined analysis, we pooled the data from all studies.

For the simulation of a long-term stroke recurrence (9), we used data taken from the control cohort of the HERMES dataset (15). The mortality of stroke patients for a particular functional outcome category (7) was determined by the hazard ratios of death at that specific mRS category, which was multiplied with the age-specific mortality obtained from U.S. life tables (18).

### Costs

Prehospital costs were not included in the simulation. Published costs from the U.S. healthcare system in U.S.$ were used to calculate cumulative costs and were adapted to 2022 values with discount rates of 3% / year: Hospital costs were derived from a nationwide analysis of acute stroke care costs by Mu et al. (13), depending on the functional outcome within the first 365 days. Expenses associated with endovascular thrombectomy (EVT) and long-term healthcare costs were approximated based on a cohort study with 428 patients (9). Acute care costs of recurrent strokes were taken from Chambers et al. (20)

### Utilities

Outcome was quantified as quality-adjusted life years (QALYs). Quality of life data were taken from a meta-analysis of multiple stroke intervention trials (19). In sensitivity analysis, we modulated all utilities in β-distributions.

### Cost-effectiveness analysis

Treatment strategies were compared in incremental costs, incremental effectiveness, and incremental cost-effectiveness ratios (ICER), which is defined by ICER = $\frac{Cost_{EVT}-Cost_{BMC}}{QALY_{EVT}-QALY_{BMC}}$.

Willingness to pay (WTP) was set to $100,000 per QALY. All costs and outcomes were discounted by 3% annually, as recommended by consensus (12).

### Sensitivity analysis

We performed deterministic and probabilistic sensitivity analyses to analyze the impact of uncertainty. The deterministic cost sensitivity revealed influences of the overall cost effectiveness after individual cost variations of + / - 25% of baseline costs.

Probabilistic sensitivity analysis was conducted in 30,000 Monte Carlo simulations by adjusting input parameters based on their probability distributions in line with previous studies (14, 21, 22).

## Results

### Cost-effectiveness analysis

In the base-case scenario, best medical care resulted in an average discounted outcome of 2.44 QALYs per patient over a lifetime horizon, while EVT resulted in 3.76 QALYs. The average discounted lifetime costs were $403,044 for best medical care and $385,726 for EVT, resulting in cost savings of $17,318 (Table 2).

While cost simulations showed discounted lifetime costs of $446,856 in RESCUE-Japan LIMIT, costs were significantly lower in simulation with ANGEL-ASPECT data ($382,679 per patient) and SELECT2 data ($325,753 per patient). The increase of effectiveness after EVT was highest in simulations using RESCUE-Japan LIMIT data (1.61 QALYs) > SELECT2 data (1.25 QALYs) > ANGEL-ASPECT data (1.12 QALYs). EVT was a cost-effective strategy in simulations with RESCUE-Japan LIMIT data and cost-saving (i.e., dominant) strategy in the other two scenarios. (Table 3).

### Probabilistic sensitivity analysis

**Figure 2:**
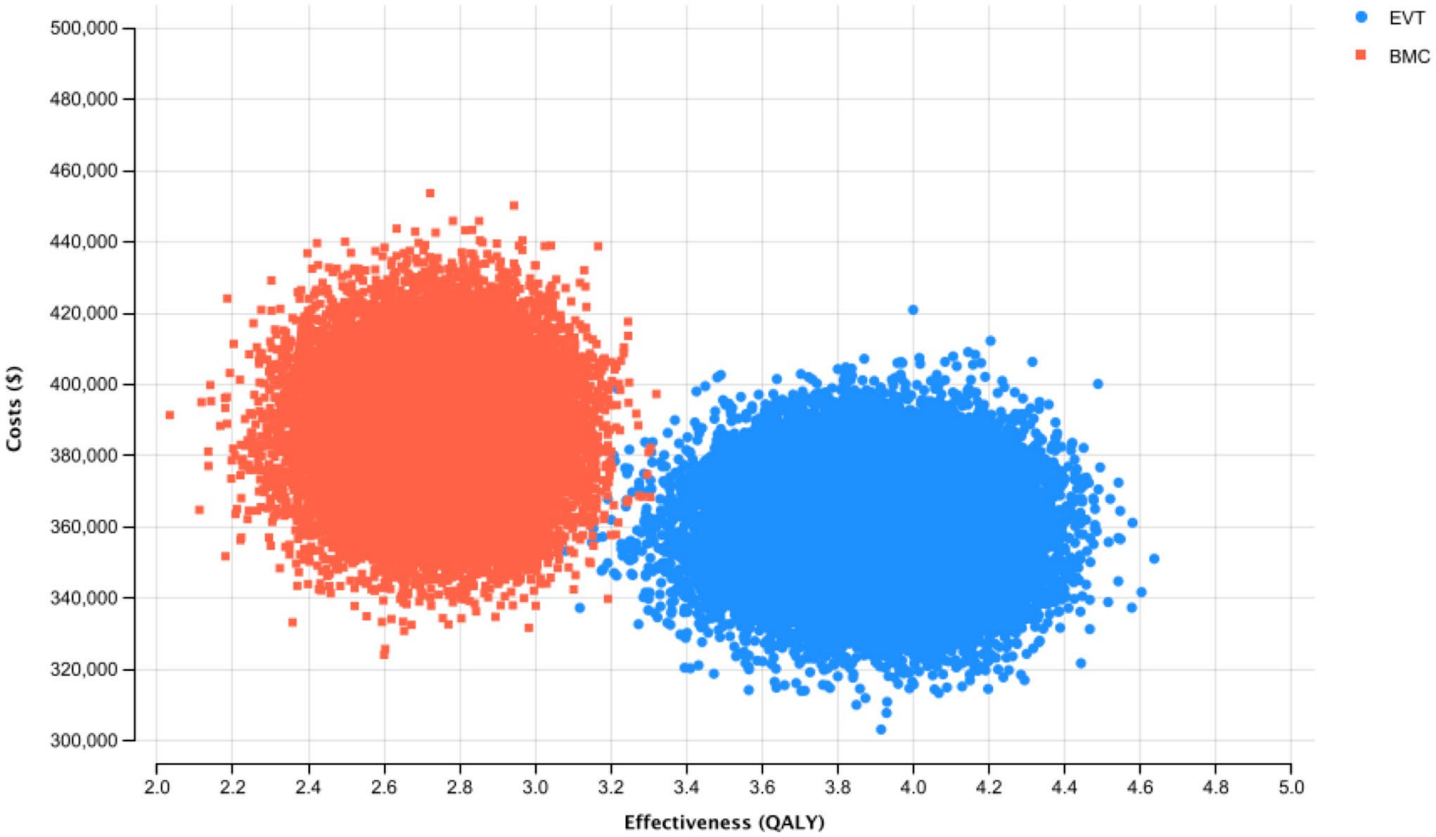
Probabilistic Sensitivity Analysis and Monte Carlo Simulation with 30,000 Iterations for the Base-Case Scenario. Thirty thousand simulations of incremental costs and outcomes of endovascular thrombectomy were compared to best medical care.

In probabilistic sensitivity analysis, EVT was cost-effective in 100% of iterations at a WTP threshold of $100,000 per QALY. No iterations showed a decrease in effectiveness while EVT was the cost-saving alternative in most iterations (Figure 2).

### Deterministic sensitivity analysis

A deterministic sensitivity analysis was conducted to consider variable costs of stroke outcomes and procedures across different healthcare systems. The cost-effectiveness of EVT was most sensitive to the costs of the treatment. However, varying the costs did not increase the ICER to positive values, so EVT remained cost-saving even with the assumption of intervention costs amounting to $21,379.

## Discussion

The conducted study is the first to evaluate the cost-effectiveness of thrombectomy in large ischemic stroke that includes the recently published international ANGEL-ASPECT and SELECT2 data.

While a previous simulation based on data from the *RESCUE-Japan LIMIT* trial demonstrated the cost-effectiveness of EVT in comparison to BMC, our simulation revealed that EVT of large vessel occlusions has cost-saving effects for health payers in a simulation conducted with data from the most recent prospective international trials. Specifically, EVT was thereby cost-saving with an improvement in effectiveness by 1.17 QALYs and a reduction of lifetime costs by $27,523 per patient. Results were most sensitive for the costs of EVT; however, an increase of 25% in intervention costs did not result in higher lifetime costs per patient than after BMC.

**Figure 3:**
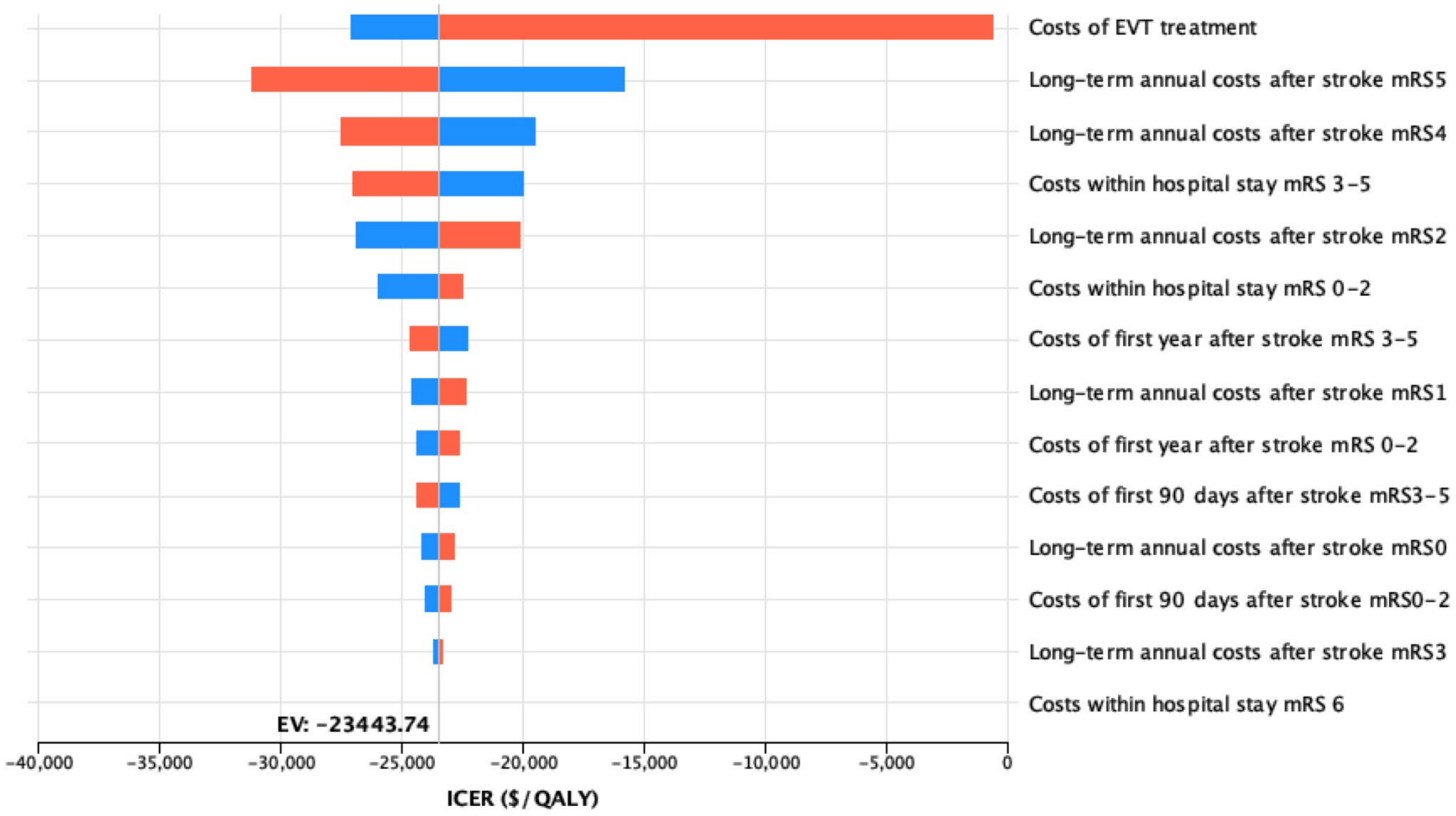
Influence of costs variations on the model outcome deterministic sensitivity analysis of the impact of all cost variations on the model outcomes upon variation by 25%. For each bar, the blue portion represents the part of the input range from the lower bound to the base case value, while the red portion represents the part of the input range from the base case value to the upper bound.

Individual simulations of the input trials revealed cost-effectiveness in the simulation with the RESCUE-Japan LIMIT data and a cost-saving effect in simulations conducted using ANGEL-ASPECT and SELECT2 data individually (Figure 3).

The cost-effectiveness of EVT in anterior circulation stroke has been shown in several previous studies, recently also in patients with mild pre-stroke disability (8, 9, 17, 21, 22). However, the cost-effectiveness of EVT in large ischemic strokes has only been investigated to a limited extent, as prospective data from international multicenter trials in this subcohort of patients has only been published very recently with the SELECT2 and ANGEL-ASPECT trials in February 2023.

Based on input data from the RESCUE-Japan LIMIT trial (3), Sanmartin et al. (10) compared lifetime costs of large ischemic stroke in the US after EVT with BMC and observed cost-effectiveness with an ICER of $16,239 / QALY. With the same input study, cost-effectiveness has also been shown for patients in different European countries with ICERs ranging from $ 2,875 / QALY in Italy to $ 5,595 / QALY in The Netherlands (23).

However, since the input study of the previously published cost simulations was only based on Japanese patients, it was necessary to repeat the cost-effectiveness analysis with the most recent studies to validate cost-effectiveness with the use of input data from the recently published international trials.

Interestingly, we discovered differences of results in dependence of the chosen input study for the simulation – likely explained by differences in outcomes.

SELECT2 (mRS 0–3 after EVT 37.9% vs. 17.9% after BMC) and ANGEL-ASPECT (mRS 0–3 after EVT 47% vs. 33.3% after BMC) showed higher outcome improvements than Rescue-Japan Limit (mRS 0–3 after EVT 31% vs. 12.7% after BMC) (24). This is of particular relevance because it shifts the simulation from a cost-effective strategy, i.e., rising costs below the established threshold of $100,000 / QUALY to a cost-saving strategy, i.e., overall reduced costs (Table 3).

Despite high initial costs, EVT may therefore save absolute costs of care in the long-term, contrary to current trends of rising costs in stroke care.

Consequently, to make EVT available to this subcohort of stroke patients would also enable cost benefits for policy makers and could compensate potential investment costs in the mid- to long-term horizon.

### Limitations

Because prospective long-term data collected at individual patient levels is not available to investigate this research question, we performed a Markov-decision-model simulation which can only simulate costs and effectiveness with several limitations:

First, results were calculated based on a Markov model with U.S. data, the generalizability of our results for other countries is limited, despite considerations of cost differences of up to 25%. Because large prospective datasets for the evaluation of mid to long-term costs of stroke in Europe are not yet available, validation of our results with European data is needed as part of future investigations.

Second, our simulation is based on simplified linear pathways for diagnostics and therapy, which results in limitations derived from the availability, quality and validity of input variables. Group imbalances and retrospective evaluations could limit the validity of model inputs. Prehospital costs were not included in our simulation, so that the absolute costs per patient are underestimated in both groups. Long-term outcomes of stroke patients were calculated based on 90-day outcome scores. However, previous trials indicated changes in stroke outcomes within the first year (25); a repetition of the analysis might be necessary when long-term outcome data of the input studies become available.

Third, the results are based on patients included in ANGEL-ASPECT, SELECT2 and Rescue Limit; the effects of triage that happen before the inclusion are therefore not represented, and differences in inclusion criteria could not be considered for the simulation. Also, population level estimates reported in this study are extrapolations based on stroke patients with an assumed age of 67 years, the average age of patients in the included trials. Diverse settings and variable practices and/or policy variations may vary the results substantially.

## Conclusion

In the conducted simulation of long-term quality of life and costs, EVT is a cost-saving (i.e., dominant) strategy in acute ischemic stroke with large ischemic cores defined by inclusion criteria of the recently published *ANGEL-ASPECT, SELECT2, and RESCUE-Japan LIMIT trials* in comparison to best medical care. Further prospective data in patients presenting with large infarcts need to be collected to validate these results.

## Data availability statement

The original contributions presented in the study are included in the article/supplementary material, further inquiries can be directed to the corresponding author.

## Author contributions

JS: Writing – original draft, Validation, Formal analysis, Conceptualization. MF: Writing – review & editing, Validation. JK: Writing – review & editing. DM: Writing – review & editing. JB: Writing – review & editing. DS: Writing – review & editing. JoR: Writing – review & editing. KD: Writing – review & editing, Formal analysis. JeR: Writing – review & editing. CZ: Writing – review & editing. TB-B: Writing – review & editing, Conceptualization. WK: Writing – review & editing, Conceptualization.
